# Excess mortality in patients with schizophrenia spectrum disorders in Malaga (Spain): A cohort study

**DOI:** 10.1017/S2045796020001146

**Published:** 2021-02-04

**Authors:** Berta Moreno-Küstner, Jose Guzman-Parra, Yolanda Pardo, Yolanda Sanchidrián, Sebastián Díaz-Ruiz, Fermin Mayoral-Cleries

**Affiliations:** 1 Departamento de Personalidad, Evaluación y Tratamiento Psicológico, Grupo GAP, Facultad de Psicología, Universidad de Málaga, Spain; 2 Department of Mental Health, University General Hospital of Malaga. Biomedical Research Institute of Malaga (IBIMA), Spain; 3 Instituto de Medicina Legal de Málaga, Spain

**Keywords:** Health outcomes, psychosis, risk factors, schizophrenia

## Abstract

**Aims:**

There is evidence that patients with schizophrenia spectrum disorders present higher mortality in comparison with the general population. The aim of this study was to analyse the causes of mortality and sociodemographic factors associated with mortality, standardised mortality ratios (SMRs), life expectancy and potential years of life lost (YLL) in patients with schizophrenia spectrum disorders in Spain.

**Methods:**

The study included a cohort of patients from the Malaga Schizophrenia Case Register (1418 patients; 907 males; average age 42.31 years) who were followed up for a minimum of 10 years (median = 13.43). The factors associated with mortality were analysed with a survival analysis using Cox's proportional hazards regression model.

**Results:**

The main causes of mortality in the cohort were circulatory disease (21.45%), cancer (17.09%) and suicide (13.09%). The SMR of the cohort was more than threefold that of the population of Malaga (3.19). The life expectancy at birth was 67.11 years old, which is more than 13 years shorter than that of the population of Malaga. The YLL was 20.74. The variables associated with a higher risk of mortality were age [adjusted hazard ratio (AHR) = 1.069, *p* < 0.001], male gender (AHR = 1.751, *p* < 0.001) and type of area of residence (*p* = 0.028; deprived urban zone *v*. non-deprived urban area, AHR = 1.460, *p* = 0.028). In addition, receiving welfare benefit status in comparison with employed status (AHR = 1.940, *p* = 0.008) was associated with increased mortality.

**Conclusions:**

There is excess mortality in patients with schizophrenia spectrum disorders and also an association with age, gender, socioeconomic inequalities and receiving welfare benefits. Efforts directed towards improved living conditions could have a positive effect on reducing mortality.

## Introduction

In general, mental disorders are strongly associated with premature mortality worldwide (Walker *et al*., 2015) and remain an important global health problem (Liu *et al*., 2017). Among them, excess mortality in schizophrenia spectrum disorders is a major public health concern that causes significant suffering in patients, families and society. Reviews of the literature on mortality in schizophrenia and psychotic disorders have found a standardised mortality ratio (SMR) of 2–4 (Saha *et al*., 2007; McGrath *et al*., 2008; Ringen *et al*., 2014; Piotrowski *et al*., 2017). A recent meta-analysis by Oakley *et al*. (2018) found a gender-pooled SMR of 3.08 for schizophrenia and psychotic disorders in the community. Likewise, a recent systematic review (Hjorthøj *et al*., 2017) found 14.5 potential years of life lost (YLL) associated with schizophrenia, with an overall weighted average life expectancy of 64.7 years, being lower for men (59.9 years) than for women (67.6 years).

Mortality in schizophrenia is associated with natural causes, mainly cardiovascular diseases (Brown *et al*., 2010; Bushe *et al*., 2010), cardiometabolic adverse events related to anti-psychotic medication (Mitchell *et al*., 2013) and other physical health comorbidities such as cancer (Crump *et al*., 2013; Laursen *et al*., 2014; Morgan *et al*., 2014). Among the unnatural causes of death, suicide is the most common (Rantanen *et al*., 2009; Brown *et al*., 2010; Laursen *et al*., 2014). Concerning sociodemographic factors, living in areas of socioeconomic disadvantage has been associated with higher mortality rates (Tsai *et al*., 2014).

In addition, the excess mortality found in schizophrenia spectrum disorders does not seem to be diminishing with the advances in diagnostic and therapeutic methods; instead, it is increasing (McGrath *et al*., 2008; Oakley *et al*., 2018). Although mortality has declined for the general population, people with schizophrenia spectrum disorders still have elevated mortality (Ösby *et al*., 2016; Westman *et al*., 2018). Lee *et al*. (2018) found that the SMR increased by 37% from pre-1970 to post-1970 studies and Bushe *et al*. (2010) found a peak in the mid-1990s. Therefore, as a public concern, excess mortality in schizophrenia remains a phenomenon that is insufficiently studied and unsolved (Piotrowski *et al*., 2017) and analyses have shown high levels of heterogeneity between regions.

Furthermore, there is a lack of studies in southern and eastern Europe on mortality in schizophrenia spectrum disorders (Gondek *et al*., 2015) and the study of possible regional differences could be informative and valuable (Galletly, 2017). The scarcity of studies in these regions makes it difficult to assess the longitudinal evolution of mortality in schizophrenia spectrum disorders in a large part of Europe and also to explore more about the different sociodemographic factors that could be influencing the so-called mortality gap. In Spain, there has been one published study estimating the characteristics of physical disease in hospitalised patients with schizophrenia, which concluded that physical disease in schizophrenia was associated with an SMR of 3.6 (Bouza *et al*., 2010). In order to evaluate the mortality in schizophrenia and related disorders, we have carried out this study based on data from a well-defined catchment area in southern Spain where a comprehensive case register operating since 2003 was developed. Hence, the purpose of the present study is to present mortality data in a cohort of patients with schizophrenia spectrum disorders in Malaga (Spain); specifically, the causes of mortality, SMR, life expectancy, YLL and possible sociodemographic factors associated with mortality were analysed.

## Methods

### Design, setting and cohort

We conducted an observational study using data from a historical cohort of the Registry of Schizophrenia of Malaga (RESMA). The RESMA was a census of patients diagnosed with schizophrenia spectrum disorders treated in any mental health services of the catchment area of the Regional University Hospital of Malaga, which covers 315 159 inhabitants. The participants were included in the RESMA between 1 January 2003 and 31 December 2006. Demographically, the area predominantly contained an urban population, with only 14% of participants living in rural areas and 9.7% living in socioeconomically deprived areas (Moreno-Küstner *et al*., 2016). The RESMA is based on healthcare data collected routinely from several psychiatric record systems merged into a unique database. Details of the baseline characteristics of the sample have been described previously (Moreno-Küstner *et al*., 2009, 2016). The cohort study included all patients with a clinical diagnosis of schizophrenia spectrum disorders (ICD-10 codes F20–F29) (World Health Organization, 2016). The subjects were followed up to 31 December 2016. The median time of follow-up was 13.43 years [interquartile range ([IQR) = 10.51–13.51 years].

We used different procedures to confirm the status of cases as alive, dead or censored. The process was performed in two stages. The first source of information was the digitised routine medical record of Andalusia: thus, when an appointment was attended after 31 December 2016 the participant was considered alive. The second source of information was the Malaga Civil Register, where deaths are registered in the INFOREG programme (computerisation of civil registries), which allows searches at the regional and national level. To corroborate the causes of mortality, the Institute of Forensic and Legal Medicine of Malaga was checked. In participants whose information had not been available in medical records, a search was performed to confirm whether the person was dead and to identify the date and cause of death. Subjects for whom no data were obtained in medical records or the Civil Registry were considered to be censored and were included in the follow-up period until the date of last contact with the health system (date of censoring).

Mortality was studied in relation to the following variables at baseline: age, gender, civil status (single, married/with partner, separated, divorced, widowed), level of education (no formal education and illiterate, primary school, secondary school, higher education), type of living arrangement (family of origin/other relatives, own family, alone, sheltered accommodation, homeless), employment status [employed, unemployed, receiving welfare benefits (because illness or ageing made it impossible for them to continue with their work), others], type of area of residence (non-deprived urban, deprived urban zone, rural, not proceed) and ICD-10 diagnosis [F20: schizophrenia, F22: persistent delusional disorders, F23: acute psychotic disorders, F25: schizoaffective disorders, others (F21: schizotypal disorder, F24: disorder of induced delusions, F28: other non-organic psychotic disorders, F29: unspecified non-organic psychosis)]. The latter variable was also categorised as schizophrenia *v*. other schizophrenia spectrum disorders in order to calculate the specific SMR, life expectancy and YLL. Natural causes of mortality were classified according to the broad categories of the ICD-10 (World Health Organization, 2016) and non-natural causes of mortality were categorised as suicides or other non-natural causes.

### Analytical methods

We calculated the mortality rate by dividing the number of observed deaths (deaths from all causes) in the cohort by the person-years at risk, multiplying by 1000 and then stratifying according to sociodemographic and diagnostic variables.

The SMRs were calculated as the ratio of the number of observed deaths in the sample and the number of expected deaths in the province of Malaga obtained by the mortality statistics of the province of Malaga in 2009 (mid-year) (National Institute of Statistic, 2009). For the calculation of SMR for suicide, the national mortality statistics in 2009 were used, as data from Malaga were not available. The expected deaths were calculated by multiplying the population in the cohort by the time in follow-up and by the specific death rate in the general population (5-year age groups), standardised by gender. The SMR was estimated for the entire cohort and by gender, age and diagnosis categories, as well as for suicide. The SMR summarises the degree to which the mortality rate is higher or lower in a specific group compared to the general population. The SMR provides an easy-to-understand approximation of the relative risk of the study cohort in comparison with the standard population.

We calculated the life expectancy of the cohort according to Chiang's method of abridged life tables, with age intervals of 5 years (Chiang and World Health Organisation, 1979). Life expectancy was calculated for the entire cohort and by gender and diagnosis categories. Life expectancy is a measure that estimates the level of mortality of a population through the years that a person is expected to live at birth under the mortality conditions of the study period. Life expectancy might be useful because it is less affected by the composition and the follow-up time of the cohort studied than SMR and is also easy to understand and interpreted.

The YLL was also calculated based on the difference between the expected age of death of deceased persons in the sample according to the National Institute of Statistics of 2009, standardised by gender and the empirical age of death of deceased individuals, divided by the number of deceased persons. The YLL reflects the years of life that the deceased would have lived if the corresponding life expectancy had been fulfilled. The YLL has advantages including the fact that it avoids arbitrary age cut-offs and assigns higher weights to the deaths at younger ages (Martinez *et al*., 2019).

Likewise, within-cohort analyses were conducted to study factors associated with mortality using Cox's proportional hazards regression models (univariate and multivariate). A univariate analysis was performed to compare the censored individuals (*N* = 99; 7.0%) and those with information on their survival until the end of the follow-up period. There was no association between the two groups, except for the type of living arrangement (*χ*^2^ = 12.405; *p* = 0.015): the censored group lived more frequently in sheltered accommodation (20% *v*. 9.7%) or alone (13.3% *v*. 8.8%) and less frequently with family or other relatives (45.6% *v*. 54.2%) or with their own family (20.0% *v*. 25.6%). The median follow-up time for non-censored individuals was 13.51 (IQR = 10.51–13.51) and for censored individuals was 9.25 (IQR = 6.36–11.79). The multivariate models were used to confounding control. The confounders were decided based on prior causal knowledge (depicting causal graphs) and following standard epidemiological confounder control rules (Shrier and Platt, 2008). The multivariate analyses that were carried out both with the original data and the missing data were imputed using Multiple Imputation by Chained Equations (MICE). The results did not vary significantly and it was decided to use the data without imputation (see online Supplementary Table S1 in the supplementary material for the results using the imputed models). The linearity of the continuous variables was evaluated using the Martingale residual graph (see online Supplementary Fig. S1 in the Supplementary Material) and the proportional hazard assumption was analysed with Cox's proportional hazards test based on Schoenfeld residuals (*cox.zph* function in R). Also, a Kaplan–Meier survival analysis was conducted. The analysis was carried out using SPSS version 19.0 and R version 3.4.1 with package survival.

## Results

In this study, 1418 patients were included. Briefly, cases were more frequently male (63.90%), middle-aged (average: 42.31; s.d.: 13.63), single (61.35%), with no formal education or primary education only (64.53%), living with their family of origin/other relatives (48.02%) and receiving welfare benefits mainly due to their severe mental illness (41.60%). Most of the cohort lives in non-deprived urban areas (72%). The majority had a diagnosis of schizophrenia (60.08%). Table 1 shows the demographic characteristics and diagnoses of the sample at baseline, the number of events (deaths), person-years of follow-up and mortality rates.

**Table 1.** Study sample description of patients with schizophrenia spectrum disorders by demographic characteristics and diagnosis at baseline

*N* = 1418, Events = 275	Total	Events (death)		Person years	Mortality rates
Variables	*N*	*N*	%	*N*	(per 1000)
Age (M, s.d.) (42.31, 13.63) (Missing data 1)					
15–29	244	18	7.38	2819	6.38
30–44	624	77	12.34	7322	10.51
45–60	384	95	24.74	4182	22.72
+ 60	165	84	50.91	1476	56.91
Gender					
Female	511	89	17.42	5730	15.53
Male	907	186	20.51	10 076	18.46
Civil Status (Missing data: 52)					
Single	870	147	16.90	9924	14.81
Married/with partner	303	65	21.45	3310	19.64
Separated/divorced/widowed	193	53	27.46	2048	25.88
Level of education (Missing data: 66)					
No formal education and illiterate	271	90	33.21	2755	32.66
Primary school	644	112	17.39	7313	15.31
Secondary school	305	43	14.10	3556	12.09
Higher education (Bachelor's degree)	132	15	11.36	1514	9.91
Type of living arrangement (Missing data: 58)					
Their parents/other relatives	681	93	13.66	7900	11.77
Own family	355	76	21.41	3941	19.28
Alone	132	31	23.48	1423	21.78
Sheltered accommodation	175	60	34.28	1777	33.76
Homeless	17	2	11.76	188	10.64
Employment status (Missing data: 65)					
Employed	228	20	8.77	2691	7.43
Unemployed	233	26	11.16	2656	9.79
Receiving welfare benefits	590	162	27.46	6455	25.10
Others	302	51	16.89	3365	15.16
Type of area of residence (Missing data: 11)					
Non-deprived urban	1021	198	19.39	11 356	17.43
Deprived urban	167	41	24.55	1831	22.39
Rural	111	16	14.41	1306	12.25
Not proceed	108	18	16.67	1211	14.86
Clinical diagnoses (ICD-10)					
F20 Schizophrenia	852	164	19.25	9622	17.04
F22 Persistent delusional disorders	233	58	24.89	2470	23.48
F23 Acute psychotic disorders	151	25	16.56	1705	14.66
F25 Schizoaffective disorders	108	14	12.96	1218	11.49
F21 Schizotypal disorder, F24 Disorder of induced delusions, F28 Other non-organic psychotic disorders and F29 Unspecified non-organic psychosis	74	14	18.92	793	18.92

### Mortality

Out of 1418 patients, 275 (19.4%) individuals died during the follow-up period, with the mortality rate being 17.40 per 1000 person-years.

The most frequent cause of mortality was circulatory system disease (21.45%, *N* = 59), followed by cancer (17.09%, *N* = 47). Non-natural causes of mortality accounted for approximately a quarter of the deaths (19.09%, *N* = 53); these were mainly due to suicide (13.09%, *N* = 36). Information regarding the causes of mortality is summarised in Table 2. Among individuals <35 years old at the beginning of follow-up, suicide was the main cause of mortality (48.38%, *N* = 15).

**Table 2.** Causes of mortality by the International Classification of Diseases 10th version

Cause of mortality *N* = 275	*N*	%
Natural causes of mortality		
Certain infectious and parasitic diseases (I)	9	3.27
Neoplasm (II)	47	17.09
Endocrine, nutritional and metabolic diseases (IV)	2	0.73
Diseases of the nervous system (VI)	7	2.54
Diseases of the circulatory system (IX)	59	21.45
Diseases of the respiratory system (X)	32	11.64
Diseases of the digestive system (XI)	7	2.54
Diseases of the genitourinary system (XIV)	2	0.73
Other natural causes of mortality not elsewhere classified	13	4.72
Non-natural causes of mortality		
Suicide	36	13.09
Other non-natural causes of mortality	17	6.18
Unknown	44	16.00

Mortality was three times higher than in the general population of Malaga (SMR = 3.189). Regarding gender, the SMR for men (3.385) was higher than for women (2.840); with regard to age groups, the SMR was especially high in the lower age ranges, and this progressively reduced: 9.323 (15–29 years), 7.670 (30–44 years), 4.325 (45–59 years) and 2.109 (>60 years). The SMR was higher in schizophrenia (3.997) compared with other schizophrenia spectrum disorders (2.534). Regarding suicide, the SMR was 22.118 [19.414 (14.112–24.716) in men and 38.002 (19.977–56.026) in women] (Table 3).

**Table 3.** Standardised mortality ratios of patients with schizophrenia spectrum disorders by gender, age intervals and diagnosis

Variables	Observed deaths	Standardised mortality ratios^a^ (CI 95%)
Total sample^b^	275	3.189 (2.925–3.452)
Gender^c^		
Men	186	3.385 (3.047–3.724)
Women	89	2.840 (2.424–3.256)
Age intervals^c^		
15–29	18	9.323 (1.274–17.371)
30–44	77	7.670 (5.181–10.159)
45–60	95	4.325 (3.617–5.033)
+ 60	84	2.109(1.939–2.279)
Clinical diagnosis^c^		
Schizophrenia	164	3.997 (3.570–4.424)
Other schizophrenia spectrum disorders	111	2.534 (2.205–2.863)
Non-natural cause of death		
Suicide^b^	36	22.119 (16.886–27.356)

a The expected deaths were calculated from the data of the National Institute of Statistics (2009).

b Standardised by age and sex.

c Standardised by age.

Life expectancy at birth was more than 13 years shorter compared to that of the population of Malaga (67.11 *v*. 80.52) and more than 14 years shorter when considering only patients diagnosed with schizophrenia (66.20 *v*. 80.52). Regarding gender, the difference in life expectancy between the cohort and the general population was higher in men (more than 13 years less: 63.94 *v*. 77.71) than in women (10 years less: 73.24 *v*. 83.28) (Table 4).

**Table 4.** Life expectancy in the province of Malaga population and in the cohort by gender and diagnosis

	Age (CI 95%)
Province of Malaga^a^	
*Total*	80.506
*Gender*	
Men	77.711
Women	83.276
Patients with diagnosis of schizophrenia spectrum disorders	
*Total*	67.105 (62.241–71.969)
*Gender*	
Men	63.940 (56.937–70.945)
Women	73.240 (58.496–87.984)
*Diagnosis*	
Schizophrenia	66.204 (57.854–74.554)
Other schizophrenia spectrum disorders	68.499 (55.866–81.133)

a Data from the National Institute of Statistics (2009).

For the entire sample, the YLL was 20.74 years. In the sample with schizophrenia, the YLL was higher (22.80 years) than in other schizophrenia spectrum disorders, except schizophrenia (17.65 years). The YLL for men was higher (21.13 years) than for women (19.90 years).

In bivariate Cox regression analysis, the following variables were associated with mortality: age (UHR = 1.062, *p* < 0.001), civil status (*p* < 0.001), level of education (*p* < 0.001), type of living arrangement (*p* < 0.001) and employment status (*p* < 0.001) (for details see Table 5). After adjustment in the multivariate analysis, the variables associated with higher mortality were age [adjusted hazard ratio (AHR) = 1.069, *p* < 0.001), male gender (AHR = 1.751, *p* < 0.001) and type of area of residence (*p* = 0.028; deprived urban zone *v*. non deprived urban zone: AHR = 1.460, *p* = 0.028). In addition, receiving welfare benefit status in comparison with employed status was associated with increased mortality (AHR = 1.940, *p* = 0.008). Detailed information on the multivariate survival analysis is shown in Table 5. According to Kaplan-Meier, the average survival was 0.986 at 1 year, 0.935 at 5 years and 0.847 at 10 years.

**Table 5.** Survival analysis of all mortality causes by Cox regression

	UHR	CI 95%	*p*	AHR	CI 95%	*p*	Proportional hazard ratios test
Age^a^	1.062	1.053–1.071	<0.001	1.069	1.059–1.078	<0.001	0558
15–29 (Reference)							
30–44	1.635	0.979–2.732	0.060	1.619	0.968–2.706	0.066	0.763
45–60	3.578	2.162–5.922	<0.001	3.850	2.323–6.383	<0.001	0.495
+ 60	9.238	5.550–15.376	<0.001	10.657	6.380–17.803	<0.001	0.713
Gender^b^							
Female (Reference)							
Male	1.189	0.923–1.531	0.180	1.751	1.350- 2.270	<0.001	0.888
Civil status^c^			0.001			0.120	
Married/with partner (Reference)							
Single	1.337	0.998–1.790	0.052	1.355	0.958–1.917	0.086	0.612
Separated/divorced/widowed	1.767	1.290–2.419	<0.001	1.427	0.976–2.088	0.067	0.564
Level of education^d^			<0.001			0.150	
No formal education and illiterate (Reference)							
Primary School	0.464	0.351–0.612	<0.001	0.764	0.344–1.067	0.073	0.721
Secondary School	0.365	0.254–0.525	<0.001	0.707	0.477–1.047	0.084	0.267
Higher education (Bachelor's degree)	0.300	0.174–0.518	<0.001	0.606	0.348–1.067	0.083	0.589
Type of living arrangement^e^			<0.001			0.090	
Original family/other relatives (Reference)							
Own family	1.650	1.219–2.235	0.001	0.794	0.564–1.118	0.187	0.153
Alone	1.863	1.241–2.797	0.003	0.973	0.630–1.501	0.900	0.964
Sheltered accommodation	2.914	2.106–4.031	<0.001	1.276	0.889–1.833	0.186	0.314
Homeless	0.919	0.226–3.728	0.906	0.410	0.100–1.681	0.216	0.580
Employment status^f^			<0.001			0.051	
Employed (Reference)							
Unemployed	1.321	0.738–2.367	0.349	1.623	0.900- 2.927	0.108	0.655
Receiving welfare benefits	3.392	2.132–5.398	<0.001	1.940	1.185–3.175	0.008	0.340
Others	2.053	1.224–3.443	0.006	1.523	0.882–2.629	0.131	0.442
Type of area of residence^g^			0.162			0.028	
Non-deprived urban (Reference)							
Deprived urban	1.290	0.921–1.806	0.138	1.460	1.042–2.047	0.028	0.224
Rural	0.699	0.420–1.164	0.169	0.743	0.446–1.236	0.252	0.055
Not proceed	0.853	0.527–1.382	0.519	0.711	0.433–1.168	0.179	0.372
Clinical diagnoses (ICD-10)^h^			0.070			0.212	
F20 Schizophrenia (Reference)							
F22 Persistent delusional disorders	1.390	1.030–1.875	0.031	0.792	0.579–1.093	0.158	0.282
F23 Acute psychotic disorders	0.860	0.565–1.310	0.483	1.241	0.811–1.898	0.320	0.823
F25 Schizoaffective disorders	0.673	0.390–1.162	0.155	0.744	0.422–1.311	0.306	0.131
F21 Schizotypal disorder, F24 Disorder of induced delusions, F28 Other non-organic psychotic disorders and F29 Unspecified non organic psychosis	1.044	0.605–1.803	0.877	1.329	0.769- 2.297	0.309	0.888

* Average survival (Kaplan-Meier) per year: 0.986; at 5 years: 0.935 and at 10 years: 0.847.

Note: UHR, unadjusted hazard ratio; AHR, adjusted hazard ratio.

a Covariates: gender and type of area of residence.

b Covariates: age.

c Covariates: age, gender, diagnosis, level of education, employment status and type of area of residence.

d Covariates: age, gender and diagnosis.

e Covariates: age, gender, level of education and diagnosis.

f Covariates: age, gender, level of education and diagnosis.

g Covariates: age and gender.

h Covariates: age and gender.

## Discussion

The main finding of this study was the corroboration of a mortality gap in patients with schizophrenia spectrum disorder compared with the general population in a wide catchment area of Spain and the association of mortality with socioeconomic disadvantages. To the best of our knowledge, this is the first study in Spain to calculate mortality indexes using a population-based register from mental health services (including mainly community care-based users and in-patients). Importantly, the study was carried out in a population in which there was a lack of information on a fundamental issue such as the mortality of patients with schizophrenia spectrum disorders in a population where the mental health care services are less developed than in the countries where mortality has been studied.

Our results indicate that the SMR was 3.189 in the cohort and 3.997 in the sample of individuals diagnosed with schizophrenia, with both values being higher than in the meta-analysis of Oakley *et al*. (2018), which found an SMR of 3.085 in schizophrenia spectrum disorders and 3.103 for studies that only included schizophrenia. The YLL found in our study was more than 5 years higher in comparison with a meta-analysis by Hjorthøj *et al*. (2017) in which the YLL was 14.5. However, despite the YLL and SMR indicating higher relative mortality in our cohort, life expectancy was higher in comparison with the value of 64.7 years reported by Hjorthøj *et al*. (2017). This apparently contradictory result could be explained by Spain having one of the most long-lived populations in the world and corroborates the mortality gap in schizophrenia in southern Spain, confirming that this population does not benefit completely from the health improvements that have occurred in our context in recent decades. Nevertheless, these results need to be replicated in extended studies. Regarding results in nearby countries, a study in France found an SMR that was very similar to that of our study (Tran *et al*., 2009), but another study in northern Italy found a lower SMR (2.17) in a population with schizophrenia (Grigoletti *et al*., 2009). The difference in SMR in the present study in comparison with the Italian study is relevant and might be explained by the limited sample size of the Italian study (4613 person-years) and the differences in socioeconomic variables between regions. It is also important to highlight that the mortality gap is higher in the younger population. Thus, one of the most concerning outcomes is that the SMR is more than nine times higher than in the general population at younger ages of 15–29 years and seven times higher at ages of 30–44 years. This could be explained by the high mortality due to suicide in the young population with schizophrenia spectrum disorders among other factors. In fact, another important result in our study is that the SMR of suicide (22.18) was higher in comparison with other studies; for example, a meta-analysis of ten studies found an SMR of 12.86 (Saha *et al*., 2007) and highlighted the need to improve treatments to prevent suicides in our environment. This elevated SMR may be influenced by the lower rates of suicide and suicidal behaviour in southern European regions, including Spain, compared with other countries in northern Europe (Castillejos *et al*., 2020). Further studies in our area are needed to explore this issue in more depth. Our study also shows that the mortality gap is higher in men than in women, as found by other studies (Laursen, 2011; Crump *et al*., 2013; Nielsen *et al*., 2013; Lesage *et al*., 2015).

Regarding natural causes of death, in accordance with our results, other studies have found that cardiovascular disease is the main cause of mortality (Ösby *et al*., 2016; Westman *et al*., 2018), highlighting the necessity to reduce cardiovascular risk in this population. Studies carried out in Spain on the physical health of a population with severe mental disorders show a high prevalence of risk factors of mortality: metabolic syndrome, tobacco consumption and unhealthy lifestyle behaviours (Manzanares *et al*., 2014; Fernández Guijarro *et al*., 2020). In general, schizophrenia spectrum disorders have been associated with unhealthy lifestyles that have a negative impact on health and also reduce access to healthcare (Kurdyak *et al*., 2012; Tsai *et al*., 2014; Jayatilleke *et al*., 2017; Gur *et al*., 2018). Likewise, results from a Spanish consensus on the physical health of patients with schizophrenia concluded that an awareness of several aspects of the physical health of this population should be increased among primary healthcare providers and specialists (Sáiz Ruiz *et al*., 2008). Concerning non-natural causes of mortality, suicide was the most frequent (13.09%) and the first cause of mortality among patients <35 years old. Our results are in accordance with those of Alaräisänen *et al*. (2009), who analysed a cohort of patients with schizophrenia, following them from 16 to 39 years and found that half of the cause of death was suicide. Likewise, Palmer *et al*. (2005) found that the suicide rate was higher in a first-episode cohort than in those followed-up later during their illness.

After controlling by confounders, several sociodemographic variables were associated with mortality: age, gender, type of area of residence and receiving welfare benefits (compared with employed status). Interestingly, our study found a relationship between mortality and participants who lived in disadvantaged socioeconomic areas. This finding is consistent with some studies suggesting that socioeconomic inequalities influence mortality and morbidity in schizophrenia spectrum disorders (Kisely *et al*., 2007; Burns *et al*., 2014). Unfavourable socioeconomic conditions are frequent in populations with serious mental illness and could affect the course of the mental disorder and influence the mortality gap (Muntaner *et al*., 2004). Also, medical advances and improvements in healthcare, which have turned illnesses that were fatal into chronic diseases, have had no relevant impact on specific subgroups, including persons with schizophrenia (Lee *et al*., 2018). Lee *et al*. (2018) suggest a parallel between socially or economically disadvantaged segments of the general population and people with schizophrenia concerning mortality gap. It is important to highlight the potential role of the recession in the results of this study as the economic crisis resulted in increased unemployment, poverty and inequality in Spain from 2008. Another important consequence of the recession was the reduction in mental health services in the catchment area (Moreno-Küstner and Masedo Gutierrez, 2017). Future studies are necessary to analyse these issues in more depth.

Finally, we found that participants receiving welfare benefits had increased mortality compared with employed participants. It is possible that receiving welfare benefits is associated with more frequent physical illnesses and higher severity of the disorder. It would be relevant to study whether employment promotion might be beneficial in preventing early mortality in this population. In summary, in accordance with Galletly (2017), socioeconomic factors cannot be ignored in the analysis of mortality outcomes.

### Strengths and limitations

To the best of our knowledge, this is the first study in Spain to evaluate mortality in schizophrenia spectrum disorders from community care settings using linked data. The study considered all patients diagnosed with schizophrenia spectrum disorders engaged with mental health services in an epidemiologically characterised cohort. Furthermore, some vulnerable groups are represented in the cohort as there is a wide range of services in the catchment area, including services for homeless individuals with severe mental disorders. A major strength of this study is that mortality data have been accurately validated using a prospective design, with a follow-up period of more than 13 years. Thus, censored data were small and comparable to similar recent studies.

However, we must consider some limitations. First, the study is affected by any errors and omissions that may exist in the clinical routine databases and there are missing data for some of the analysed variables. Second, a source of bias could be the use of a prevalence rather than an incidence cohort, as most participants were middle-aged during the index period and had already survived the period of highest mortality. This is important because the follow-up was related to the earliest date of registry in the RESMA, but not necessarily the date of disease onset or earliest contact with a mental health service. Third, we have only measured potential risk factors for mortality at baseline, despite some variables being time-varying. Fourth, the diagnosis in the RESMA is not likely to be as accurate as the information obtained in a more structured research setting. However, studies have shown strong agreement between clinical diagnosis in registers and research settings (Jakobsen *et al*., 2005; Moreno-Küstner *et al*., 2016). The clinical diagnosis of a participant in the study was checked by senior psychiatrists in direct contact with participants. Fifth, the recruitment process missed individuals who avoided service contact during the index period, but, as published previously (Moreno-Küstner *et al*., 2016), very few patients with schizophrenia spectrum disorders are not in contact with public mental health services. Sixth, the YLL is not always calculated based on regional statistics and the different methodologies could make a comparison between studies problematic. Also, it is difficult to compare the SMR with other regions or countries with different age compositions and SMR of suicide is limited by the sample size of the cohort. Last, the generalisation of our analysis needs to be evaluated further, as the findings clearly refer to the population who had contacted mental health services within the specified period.

## Conclusions and clinical implications

In summary, this study showed that individuals suffering from schizophrenia spectrum disorders had higher mortality than the general population. The study provides detailed information about mortality: the SMR was more than 3, the YLL was more than 20 and life expectancy at birth was 67.11 years. Finally, several characteristics, such as age, being male, living in socioeconomically deprived urban areas and receiving welfare benefits, were associated with mortality.

Our results imply that urgent studies are required in Spain and southern Europe to determine factors associated with premature mortality in schizophrenia spectrum disorders and how they might be reduced in our environment. Future studies should explore the relative contribution to the mortality of, for example, medication and substance use, among others. Our findings support the necessity to implement strategies to improve the healthcare of this population and increase efforts to prevent suicide. Actions directed to improving living conditions, socioeconomic integration and the quality of health services could be beneficial in reducing mortality.

## Data Availability

The data are available under request to the authors.

## Supplementary material

For supplementary material accompanying this paper visit https://doi.org/10.1017/S2045796020001146. click here to view supplementary material
