# Supplementary material for: Excess mortality in patients with schizophrenia spectrum disorders in Malaga (Spain): A cohort study
Source: Epidemiol Psychiatr Sci. 2021 Feb 4;30:e11. doi: 10.1017/S2045796020001146 (PMC8057505; doi:10.1017/S2045796020001146)
Supplement: Supplementary file 1 [file S2045796020001146sup001.docx]

Table S1. Adjusted regression Cox models with imputed data.

|  | AHR | CI 95% | p |
| --- | --- | --- | --- |
| Age  15-29 (Reference)  30-44  45-60  +60 | 1.066  1.591  3.735  10.128 | 1.056-1.075  0.954-2.654  2.259-6.176  6.072-16.895 | <0.001  0.075  <0.001  <0.001 |
| Gender  Female (Reference)  Male | 1.730 | 1.335- 2.241 | <0.001 |
| Civil Status  Married/with partner (Reference)  Single  Separated/divorced/widowed | 1.300  1.358 | 0.917-1.841  0.934-1.976 | 0.140  0.109 |
| Level of education  No formal education and illiterate (Reference)  Primary School  Secondary School  Higher education (Bachelor’s degree) | 0.758  0.711  0.601 | 0.563-1.020  0.483-1.047  0.349-1.036 | 0.067  0.084  0.067 |
| Type of living arrangement  Original family/other relatives (Reference)  Own family  Alone  Sheltered accommodation  Homeless | 0.815  1.021  1.253  0.435 | 0.579-1.148  0.670-1.556  0.869-1.807  0.117-1.624 | 0.242  0.922  0.227  0.215 |
| Employment status  Employed (Reference)  Unemployed  Receiving welfare benefits  Others | 1.533  1.902  1.501 | 0.852- 2.760  1.161-3.116  0.851-2.649 | 0.154  0.011  0.160 |
| Type of Area of residence  Non deprived Urban (Reference)  Deprived Urban  Rural  Not proceed | 1.461  0.737  0.736 | 1.043-2.048  0.444-1.223  0.459-1.183 | 0.028  0.238  0.205 |
| Clinical diagnoses (ICD-10)  F20 Schizophrenia (Reference)  F22 Persistent delusional disorders  F23 Acute psychotic disorders  F25 Schizoaffective disorders  F21Schizotypal disorder,F24 Disorder of induced delusions.,F28 Other non-organic psychotic disorders and F29 Unspeciﬁed non organic psychosis | 0.793  1.238  0.799  1.327 | 0.577-1.089  0.811-1.890  0.605- 1.057  0.786-2.240 | 0.153  0.325  0.423  0.311 |

Figure S1. Martingale residuals graph.
